# Frequency-Stable Low-Threshold SBS-OEO for Precision Temperature Sensing in Electromagnetically Harsh Environments

**DOI:** 10.3390/s25196166

**Published:** 2025-10-05

**Authors:** Yichao Teng, Mingyuan Yang, Li Han, Jixuan Wang, Guanbo Liu

**Affiliations:** 1 College of Meteorology and Oceanography, National University of Defense Technology, Changsha 410073, China; tengyichao@nudt.edu.cn (Y.T.);; 2 Department of General Education, Changsha University of Science and Engineering, Changsha 410004, China

**Keywords:** optoelectronic oscillator, temperature sensing, electromagnetic immunity, stimulated Brillouin scattering, frequency-stable

## Abstract

**Highlights:**

**What are the main findings?**

- Threshold Reduction and Environmental Robustness
- Introduced a Faraday rotation mirror (FRM) to enable dual-pass SBS interaction, reducing the SBS power threshold by 2.7 dB compared to conventional systems.
- The FRM’s polarization rotation inherently suppresses polarization-induced fluctuations and vibration noise, eliminating the need for active polarization control.
- Record-Breaking Performance
- Sensitivity: 1.0609 MHz/°C (R^2^ = 0.999) over a 67 °C range (30–97 °C).
- Stability: ±0.004 °C long-term precision, surpassing existing SBS-OEO schemes.
- Power Efficiency: Operates at 13.6 dBm pump power.

**What is the implication of the main finding?**

- For threshold reduction and environmental robustness: The dual-pass SBS interaction enabled by FRM provides a feasible technical path to lower the energy consumption of SBS-based systems, while the inherent suppression of polarization fluctuations and vibration noise simplifies the system structure (avoiding complex active polarization control modules), reducing the difficulty of system integration and maintenance costs.
- For record-breaking performance: The high sensitivity (1.0609 MHz/°C, R^2^ = 0.999) over a 67 °C range allows the system to achieve high-precision temperature sensing in scenarios requiring wide-temperature-range monitoring (e.g., industrial equipment thermal management, environmental temperature tracking). The ±0.004 °C long-term precision surpasses existing SBS-OEO schemes, making it suitable for high-stability application scenarios such as precision instrument calibration and quantum sensing auxiliary temperature control. Additionally, the low pump power (13.6 dBm) enhances the system’s portability and energy efficiency, laying a foundation for its practical application in field detection or battery-powered devices.

**Abstract:**

In this research, precision temperature sensing for electromagnetically harsh environments was achieved utilizing a low-threshold frequency-stable optoelectronic oscillator (OEO) leveraging stimulated Brillouin scattering (SBS). The sensing mechanism relied on the temperature-dependent frequency shift in the SBS-induced notch filter. By embedding this filter in the OEO feedback loop, the oscillator’s output frequency was locked to the difference between the optical carrier frequency and the SBS notch center frequency. The temperature variations were translated into microwave frequency shifts through OEO oscillation, which was quantified with heterodyne detection. To suppress environmental perturbations, a Faraday rotation mirror (FRM) was integrated at the fiber end, creating a dual-pass SBS interaction that simultaneously enhanced the vibration immunity and reduced the SBS power threshold by 2.7 dB. The experimental results demonstrated a sensitivity of 1.0609 MHz/°C (R^2^ = 0.999) and a long-term stability of ±0.004 °C. This innovative scheme demonstrated significant advantages over conventional SBS-OEO temperature sensing approaches, particularly in terms of threshold reduction and environmental stability enhancement.

## 1. Introduction

Temperature sensing plays a pivotal role in industrial automation and environmental monitoring [1]. Fiber-optic temperature sensing, which is characterized by its exceptional immunity to electromagnetic interference, a key advantage for operations in harsh environments, along with high stability and sensitivity, has garnered increasing attention in both the scientific and industrial communities [2]. Current research encompasses diverse fiber-based architectures, including Fiber Bragg Grating (FBG) sensors, Raman scattering-based distributed systems, and multi-longitudinal-mode fiber laser configurations [3,4,5,6,7,8,9,10]. It is noteworthy that other distributed scattering mechanisms, namely Rayleigh and Brillouin scattering, also form the basis of powerful sensing techniques. Rayleigh-based systems (e.g., φ-OTDR) are highly sensitive to vibration and acoustic waves, while Brillouin-based systems (BOTDR/BOTDA) excel in simultaneous distributed temperature and strain sensing over long distances (tens of kilometers) with high accuracy [11].

To overcome these constraints, microwave photonic (MWP) technology has emerged as a transformative solution. This methodology maps optical spectral shifts to microwave frequency variations, enabling measurement rates exceeding 10 kHz and frequency resolution down to the sub-Hz level [12,13], which translates to a potential temperature resolution orders of magnitude higher than that of OSA-based approaches. A notable demonstration of this high-resolution capability can be found in the work of Pan et al. [14], who developed a microwave-photonics-based optical vector analyzer that achieved a record 50 kHz resolution (equivalent to 0.4 fm at 1550 nm), representing a 4000-fold improvement over the resolution limits of traditional OSA-based approaches. Among various MWP interrogators, the optoelectronic oscillator (OEO) has been demonstrated to be an effective methodology for high-precision sensing. Recent advancements have exploited the OEO’s frequency sensitivity to temperature and polarization, and subtle optical wavelength shifts (pm-scale) were transduced into microwave frequency deviations (kHz-scale) through opto-electro-mechanical coupling mechanisms [15].

In recent years, optoelectronic oscillator-based temperature sensing has evolved based on multiple methodologies with distinct operational principles. Early implementations utilized cavity length modulation, as exemplified by Zhejiang University’s 2014 system, achieving 43.91 kHz/°C sensitivity via thermo-optic effects, although this system was fundamentally limited by the low thermal response of silica [16]. Subsequent progress integrated stimulated Brillouin scattering (SBS) effects, as demonstrated by Chongqing University’s 2019 configuration, attaining a 1.00745 MHz/°C sensitivity through Brillouin frequency shifts. However, this configuration required a pump power exceeding 20 dBm to overcome the SBS thresholds [17]. Vernier-effect architectures emerged as a breakthrough, with Nanjing Normal University’s 2020 dual-loop design amplifying sensitivity to 210.25 kHz/°C via free spectral range matching [18], while Zhejiang University’s 2024 adaptive sampling method achieved 222.84 kHz/°C sensitivity utilizing algorithmic signal processing [19]. Despite these advancements, critical barriers persist. Conventional SBS-based systems demand unsustainable optical power levels above 20 dBm. Vernier configurations impose sub-50 ppm frequency tolerances on intracavity components. FBG-integrated solutions face scalability challenges from complex grating manufacturing. All of the existing methodologies necessitate stringent polarization control to suppress polarization-induced frequency drift, which severely constrains their applicability in environments with mechanical vibrations or electromagnetic interference. Current research efforts prioritize photonic integration techniques and polarization-diverse architectures to simultaneously address power consumption, fabrication costs, and environmental robustness while maintaining sub-millikelvin resolution.

The persistent limitations of conventional SBS-based temperature sensors—which are characterized by excessive optical power thresholds and vulnerability to mechanical vibrations—have motivated the development of a novel sensing paradigm. In this work, a temperature-sensing architecture was established through the synergistic integration of a low-threshold frequency-stable SBS and OEO. By engineering dual-pass SBS interaction via a Faraday rotation mirror (FRM), we achieved simultaneous 2.7 dB threshold reduction compared with single-pass configurations. The core innovation lay in locking the OEO’s microwave output frequency to the spectral offset between the optical carrier and the SBS-induced notch filter, whose temperature-dependent shift enabled real-time quantification through heterodyne detection. Experimental validation confirmed 1.0609 MHz/°C sensitivity (R^2^ = 0.999) with ±0.004 °C long-term precision, which addressed critical gaps in current SBS-OEO systems regarding power efficiency and environmental robustness. These advancements position the proposed architecture as a transformative solution for reliable precision temperature monitoring in electromagnetically harsh industrial environments while simultaneously establishing a framework for future extensions to multi-parameter sensing.

## 2. Operation Principle

**Figure 1:**
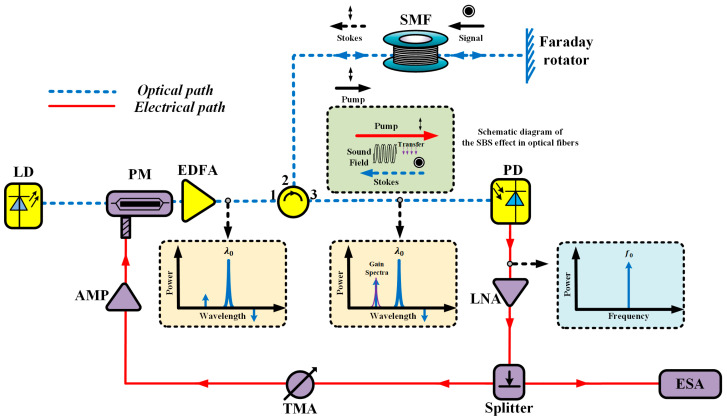
The setup for temperature sensing via a low-threshold frequency-stable SBS-based optoelectronic oscillator. LD: laser diode; PM: phase modulator; EDFA: erbium-doped fiber amplifier; OC: optical circulator; SMF: single-mode fiber; FRM: Faraday rotation mirror; PD: photodetector; LNA: low-noise amplifier; TMA: tunable microwave attenuator; AMP: microwave amplifier; ESA: electrical spectrum analyzer.

The experimental setup for the low-threshold, frequency-stable temperature sensing method based on an SBS OEO is illustrated in Figure 1. The system consisted of a laser diode (LD), phase modulator (PM), erbium-doped fiber amplifier (EDFA), optical circulator (OC), single-mode fiber (SMF), Faraday rotation mirror (FRM), photodetector (PD), low-noise amplifier (LNA), electrical power splitter, tunable microwave attenuator (TMA), microwave amplifier (AMP), and electrical spectrum analyzer (ESA). Continuous-wave light from the LD was phase-modulated by the PM and driven with microwave signals, then amplified through the EDFA before propagating along the SMF via the OC. The FRM enabled double-pass Brillouin interaction by reflecting the optical wave through the SMF twice, which effectively reduced the SBS threshold while mitigating polarization-induced fluctuations. The backward-propagating Brillouin-scattered light interacted with the forward-propagating pump wave and generated a narrowband microwave photonic filter response that was converted into electrical signals via the PD. This microwave signal underwent amplification through cascaded LNA and AMP stages, with the TMA providing closed-loop gain control to sustain stable oscillation. The system’s frequency–temperature correlation was ultimately characterized by the ESA utilizing spectral analysis of the sustained microwave oscillations.

The optical signal, after phase modulation and EDFA amplification, was directed through the optical circulator into the temperature-sensing SMF. The input laser beam served as the pump light to initiate the first SBS interaction, generating Stokes light. The pump light was then reflected by the Faraday mirror and re-entered the SMF, enabling a secondary SBS interaction with subsequent pump waves. Temperature variations in the SMF altered the frequency difference between the pump and Stokes light, corresponding to a measurable shift in the Brillouin frequency. The modulated optical signal was routed back through the circulator to the photodetector for conversion into a microwave signal. This signal was first amplified by the Low-Noise Amplifier (LNA) and then divided by a 50/50 RF power splitter. 50% of the power was tapped off and directed to the Electrical Spectrum Analyzer (ESA) for real-time monitoring and frequency analysis. The remaining 50% was fed back into the OEO feedback loop, where it was conditioned by the Tunable Microwave Attenuator (TMA) and Microwave Amplifier (AMP) to sustain stable oscillation. Within the operational range, the Brillouin frequency shift exhibited a near-linear dependence on the ambient temperature of the sensing element composed of the SMF and Faraday mirror assembly. This correlation enabled precise temperature quantification via the tracking of the oscillator’s frequency deviation. Changes in the oscillation frequency directly corresponded to temperature fluctuations through a deterministic proportionality constant derived from the system’s optoelectronic response characteristics. The relationship between the oscillation frequency variation $∆f_{osc}$ and temperature change ∆T can be described as [20,21].

(1) $$∆f_{osc}=C^{T}∆T$$

The temperature coefficient of the sensing fiber is denoted by $C^{T}$. The temperature coefficient for G.652 fiber is typically in the range of 1–2 MHz/°C, with a specific value of 1.06 MHz/°C observed in standard G.652 variants, dependent on specific fiber parameters [22]. The manifestation of SBS requires critical energy conditions to be met, for which significant SBS effects become observable only when the incident optical power reaches or exceeds this threshold. For a 5 km G.652 fiber, the theoretical SBS power threshold is calculated as approximately 9.1 dBm under ideal conditions. However, practical implementations exhibit elevated thresholds that exceed 15 dBm, which is primarily due to spectral broadening from the finite laser linewidth and additional attenuation-induced losses in the fiber link.

In the configuration shown in Figure 1, the Faraday rotation mirror reflected the pump light back into the SMF. This created a double-pass interaction geometry in which the retroreflected pump wave coherently overlapped with subsequent forward-propagating pump waves. This bidirectional coupling effectively doubled the effective optical pump power within the fiber (neglecting FRM insertion loss), thereby inducing a cumulative power buildup that amplified the Brillouin interaction efficiency. This coherent enhancement reduced the SBS power threshold by 3 dB compared with conventional single-pass configurations since the intensity-dependent nonlinear process benefited from the prolonged photon–phonon interaction length. The threshold reduction enabled stable operation at lower input power levels while preserving the temperature-dependent Brillouin frequency shift characteristics.

The polarization characteristics of the FRM’s input/output optical signals were analyzed by considering its unique polarization rotation mechanism. With the assumption that the Jones vector of the input polarized light was represented as a generic polarization state, the FRM introduced a 45° polarization rotation through the Faraday effect during forward propagation, the retroreflected light underwent an additional 45°rotation in the reverse direction, resulting in a total 90° polarization transformation. This orthogonal polarization alignment between the forward and backward propagating waves effectively canceled polarization-dependent loss (PDL) by ensuring that the output polarization state remained fixed relative to the input and was independent of environmental perturbations. The non-reciprocal nature of the Faraday rotation guaranteed that the polarization state after double-pass transmission became orthogonal to the initial input, thereby eliminating polarization fading and ensuring system stability through passive polarization scrambling suppression.

## 3. Experimental Results

**Figure 2:**
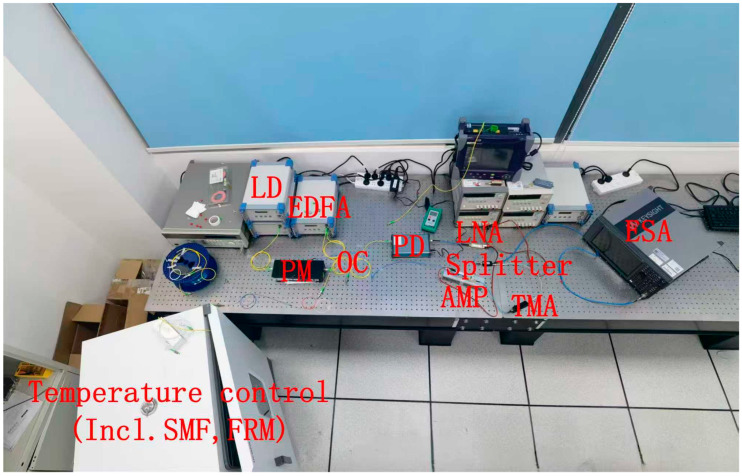
The actual experimental setup is based on the structure depicted in Figure 1.

Figure 2 shows the actual experimental setup based on the structure depicted in Figure 1. The experimental setup utilized a 5 km spool of G.652 single-mode fiber as the sensing element. The 5 km length of the sensing fiber was selected to optimize the oscillator’s frequency stability. The double-pass configuration enabled by the FRM results in an effective cavity length of 10 km. This length produces a mode spacing of approximately 20 kHz, which effectively suppresses adjacent modes and yields superior phase noise performance. Furthermore, this length provides a sufficient Brillouin gain medium to achieve a low SBS power threshold, fulfilling the primary objective of this work. The setup was driven by a tunable laser source (1529–1563 nm wavelength range) with a maximum output power of 13 dBm (20 mW). The EDFA stage provided 27 dB optical gain with a 5.8 dB noise figure and 19 dBm saturated output power, while the phase modulation was implemented using an iXblue MPZ-LN-20 modulator (Saint Germain en Laye, France) with a 20 GHz bandwidth. Detection was performed using a U2T BPRV2125AM (Berlin, Germany) photodiode featuring a 31 GHz bandwidth and 0.5 A/W responsivity, complemented by a Faraday rotation mirror with 45° polarization rotation to ensure polarization insensitivity. The OEO loop maintained 35 dB RF gain through cascaded amplification stages. The sensing fiber was placed in a high-precision thermal chamber (LiChen Instruments LC-HN-60BSG Shaoxing, China), which provided stable temperature control from 5 °C to 100 °C with 0.1 °C resolution and an overall system accuracy of ±0.5 °C. Key experimental components and their parameters are summarized in Table 1.

**Figure 3:**
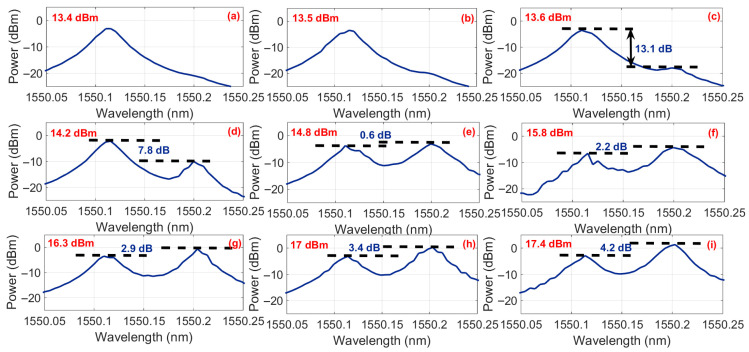
The optical spectral characteristics with FRM integration for varying EDFA output power levels. (**a**) Below threshold regime (13.4 dBm): Only pump wave observed at 1550.00 nm. (**b**) Below threshold regime (13.5 dBm). (**c**) Threshold point (13.6 dBm): Emergence of Stokes light at 1549.91 nm (0.09 nm offset), with pump dominance of 13.1 dB. (**d**) Threshold point (14.2 dBm): pump dominance of 7.8 dB. (**e**) Threshold point (14.8 dBm). (**f**) Threshold point (15.8 dBm). (**g**) Power inversion (16.3 dBm): Stokes light intensity exceeding pump by 2.9 dB, confirming full SBS energy transfer. (**h**) Power inversion (17 dBm). (**i**) Power inversion (17.4 dBm).

Figure 3 presents the optical spectra for varying EDFA output power levels with the FRM integrated into the system. Below the critical threshold of 13.6 dBm EDFA output power, no Stokes light generation was observed, confirming the absence of SBS. As the EDFA power exceeded 13.6 dBm, the onset of SBS became evident with Stokes light emerging at a wavelength offset of 0.09 nm (corresponding to an ~11 GHz frequency shift at 1550 nm) from the pump wave. The measured Stokes power reached −17.3 dBm, while the pump maintained a 13.1 dB power dominance over the Stokes component. At the injected optical power of 15.8 dBm, the power difference between the pump and Stokes waves is minimized.

**Figure 4:**
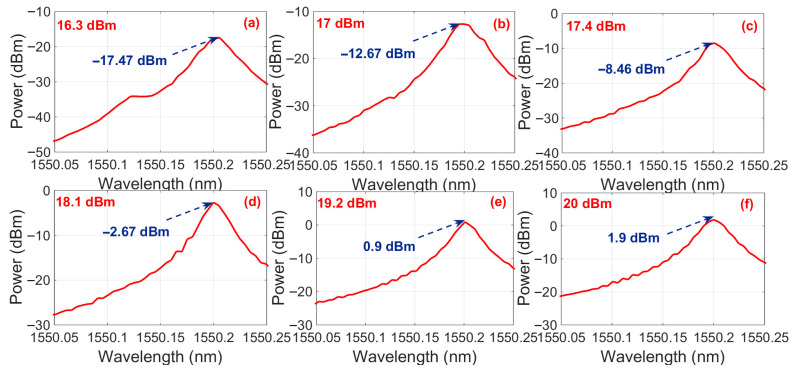
The optical spectral characteristics without FRM integration for varying EDFA output power levels. (**a**) Threshold point (16.3 dBm). (**b**) Power inversion (17 dBm). (**c**) Power inversion (17.4 dBm). (**d**) Power inversion (18.1 dBm). (**e**) Power inversion (19.2 dBm). (**f**) Power inversion (17.4 dBm).

Figure 4 demonstrates the optical spectral characteristics without FRM integration for varying EDFA output power levels. At the 16.3 dBm EDFA output power, the Stokes light intensity reached −17.47 dBm, confirming SBS generation for the threshold criteria defined in Figure 3c and thereby establishing 16.3 dBm as the SBS power threshold for the non-FRM configuration. A progressive increase in the EDFA output power beyond this threshold amplified the Stokes light intensity, achieving 1.9 dBm at the 20 dBm pump power.

**Figure 5:**
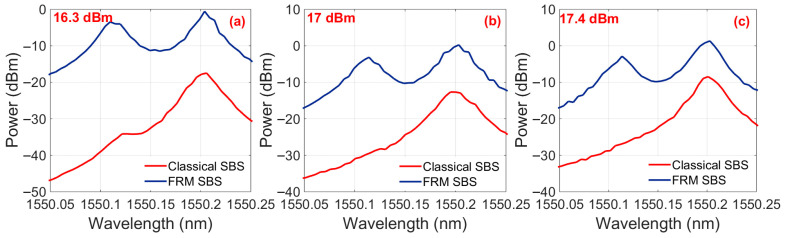
Comparative output optical spectra of FRM-enhanced SBS versus conventional SBS configurations for varying EDFA output power. (**a**) 16.3 dBm. (**b**) 17 dBm. (**c**) 17.4 dBm.

**Figure 6:**
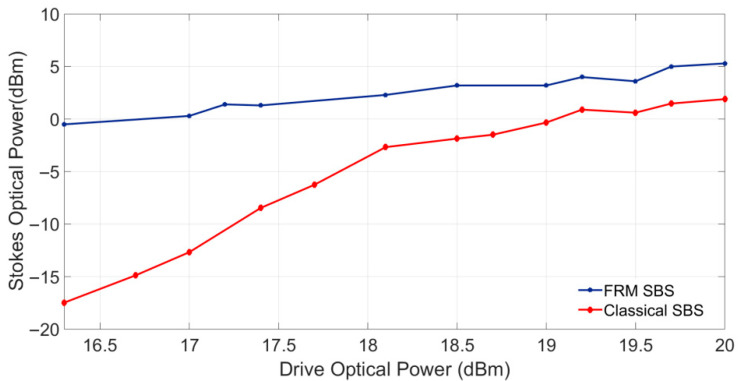
Stokes optical power versus pump power characteristics with and without FRM integration.

Figure 5 illustrates a comparison of the output optical spectra of FRM-enhanced SBS versus conventional SBS configurations for fixed EDFA output power, demonstrating the Faraday rotation mirror’s capability to boost Stokes signal power through double-pass interaction enhancement. The measured Stokes optical power versus pump power characteristics (Figure 6) revealed critical performance enhancements enabled by FRM integration. At the 18.1 dBm pump power, the FRM-enhanced SBS configuration generated 2.3 dBm Stokes power, exceeding the classical SBS output (−2.7 dBm) by 5 dB.

**Figure 7:**
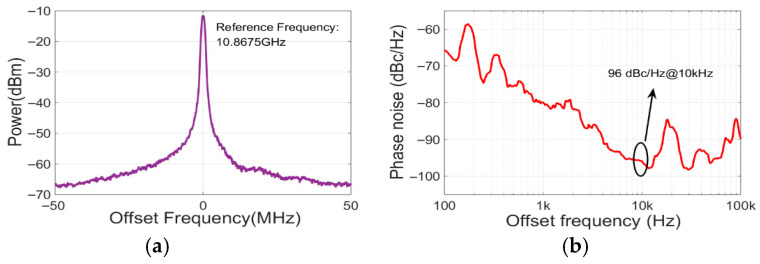
The system’s output microwave signal characteristics and phase noise performance. (**a**) Spectral profile at 30 °C: Dominant oscillation peak at 10.8675 GHz with the power level of −10 dBm (Span: 100 MHz, RBW: 9.1 kHz). (**b**) Phase noise curve.

For the temperature sensing measurements presented hereafter, the EDFA output power was set to approximately 15.8 dBm. This level was chosen to operate well above the SBS threshold to ensure a high signal-to-noise ratio for the OEO, while remaining below the power inversion point to maintain oscillation stability. Figure 7 presents the system’s output microwave signal characteristics and phase noise performance. Figure 6 displays the spectral profile measured at 30 °C with a 100 MHz span and 9.1 kHz resolution bandwidth. The profiles show a dominant oscillation frequency of 10.8675 GHz at the −10 dBm power level, which is consistent with the theoretical predictions. Figure 6 details the phase noise characteristics. In the figure, the curve exhibits an overall descending trend with pronounced peaks in the 100 Hz to 1 kHz offset frequency region. Critical performance markers include a phase noise level of −96 dBc/Hz at the 10 kHz offset. The observed 20 kHz mode spacing corresponds to the effective 10 km fiber length (5 km spool × 2 passes through Faraday mirror reflection), with its phase noise elevation attributed to polarization-dependent interference in the extended cavity configuration. The primary phase noise limitation stemmed from the 10 MHz laser linewidth that was employed. The relatively large linewidth of the laser source significantly degraded the phase noise of the output signal. Employing a narrow-linewidth laser would substantially enhance the phase noise performance.

**Figure 8:**
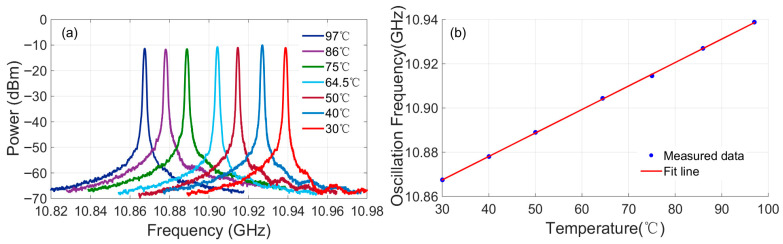
The temperature-dependent frequency response of the OEO system. (**a**) Overlaid microwave spectra. (**b**) Linear regression.

Figure 8 characterizes the temperature-dependent frequency response of the OEO system. By applying controlled temperature variations to the sensing single-mode fiber (SMF), the resulting oscillating microwave signals were captured using an electrical spectrum analyzer (ESA). Figure 8a displays overlaid spectra of the generated microwave signals across the tested temperature range. Figure 8b presents the corresponding oscillation frequency as a function of temperature. The data reveal a linear increase in the oscillation frequency from 10.8675 GHz at 30 °C to 10.9388 GHz at 97 °C. Linear regression analysis yields the following relationship:

(2) $$f_{osc}(MHz)=1.0609(MHz/{}^{\circ}C)\times T({}^{\circ}C)+10835.66(MHz)$$

with a correlation coefficient (R^2^) of 0.999. This corresponded to a temperature sensitivity of 1.0609 MHz/°C. It is important to note that while the fundamental sensing capability of the SMF spanned the range of 30–97 °C. The overall system accuracy was limited by the calibration uncertainty of the commercial thermal chamber (LC-HN-60BS) used as the reference standard.

**Figure 9:**
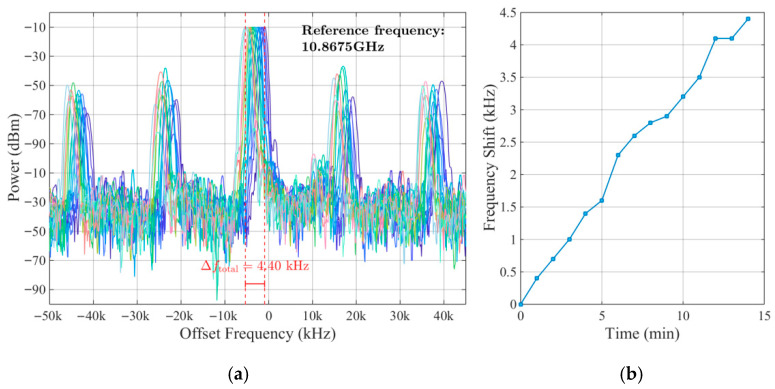
Long-term frequency precision characterization of the OEO temperature sensing system. (**a**) Time-domain frequency drift. (**b**) Center frequency variation over 14 min at one-minute intervals.

Figure 9a illustrates the frequency drift characteristics over time. Measured at one-minute intervals across a 14 min period, the center frequency exhibited a consistent leftward drift, accumulating a total shift of 4.40 kHz. This corresponded to a highly precise temperature error rate of 0.004 °C. Figure 9b presents this temporal evolution as a line graph. The figure clearly depicts the gradual frequency decrease of 4.40 kHz over the observation window.

Our solution (FRM) demonstrated significant advantages for key performance metrics. It offered unparalleled frequency stability (4.4 kHz) and top-tier measurement precision (0.004 °C) while maintaining low system complexity. Compared with schemes with similar precision, our scheme provided a more practical temperature range (67 °C), achieving an excellent balance among ultra-high precision, stability, complexity, and practicality. The results of this comparison with other schemes are summarized in Table 2.

## 4. Conclusions

In summary, we proposed and experimentally demonstrated a temperature-sensing system capable of operation in electromagnetically harsh environments based on a low-threshold frequency-stable SBS optoelectronic oscillator. By integrating a Faraday rotation mirror (FRM) to create a dual-pass SBS interaction within the OEO feedback loop, the new architecture significantly reduced the SBS power threshold. Compared with the conventional SBS-OEO structure, this reduction was 2.7 dB. The system also enhanced immunity to environmental perturbations. This improvement specifically targeted polarization-induced fluctuations and vibrations. The system transduced the temperature-dependent SBS frequency shift into a measurable microwave oscillation frequency. Experimental validation confirmed a high sensitivity of 1.0609 MHz/°C (R^2^ = 0.999) over a 67 °C range and exceptional long-term stability of ±0.004 °C. The ±0.004 °C stability, combined with inherent electromagnetic immunity, will enable deployment in electromagnetically harsh precision manufacturing environments (e.g., semiconductor thermal control) previously unattainable with conventional electronic sensors.

## Figures and Tables

**Table 1:** Key experimental components and their parameters.

Component	Model/Specifications	Key Parameters/Set Value	Note/Vendor
Laser Diode (LD)		Wavelength: 1550.12 nm; Output Power: 13 dBm; Linewidth: 10 MHz	Main optical source
Phase Modulator (PM)	MPZ-LN-20 (iXblue) Saint Germain en Laye, France	Bandwidth: 20 GHz; Vπ: ~3.5 V	For sideband generation
Erbium-Doped Fiber Amplifier (EDFA)	-	Small-Signal Gain: 27 dB; Noise Figure: 5.8 dB; Saturated Output Power: 19 dBm; Operating Point: 13.6–16.3 dBm	Provides SBS pump power
Photodetector (PD)	BPRV2125AM (U2T) Berlin, Germany	Bandwidth: 31 GHz; Responsivity: 0.5 A/W	O/E conversion
Single-Mode Fiber (SMF)	G.652.D	Length: 5 km; Attenuation: ~0.2 dB/km; Brillouin Frequency Shift: ~10.867 GHz @1550 nm, 30 °C	Sensing medium
Faraday Rotation Mirror (FRM)	-	Polarization Rotation: 45°; Insertion Loss: <0.5 dB	Enables double-pass, suppresses polarization fading
Low-Noise Amplifier (LNA)	-	Gain: 20 dB; Noise Figure: <3 dB	RF signal pre-amplification
Microwave Amplifier (AMP)	-	Gain: 15 dB	Provides OEO loop gain
Tunable Microwave Attenuator (TMA)	-	Attenuation Range: 0–30 dB	Finely tunes loop gain for stable oscillation
Thermal Chamber	LC-HN-60BS (LiChen) Shaoxing, China	Control Range: 5–100 °C; Set Temperature: 30–97 °C; Stability: ±0.1 °C	Provides a temperature environment
Electrical Spectrum Analyzer (ESA)	N9020B (Keysight) Santa Rosa, US	Analysis Bandwidth: 10 Hz–26.5 GHz; Resolution Bandwidth (RBW): 9.1 kHz	Monitors oscillation frequency

**Table 2:** Performance comparison of different OEO-based temperature sensing techniques.

Fundamental Principles	Technology Approach	Sensitivity	Freq. Stability	Meas. Time	SBS Threshold Power	Fiber Length	Precision	Range	Complexity
Fiber Property	Injection locking [16]	43.91 kHz/°C	5.4 kHz	60 s	-	8 m	0.12 °C	220 °C	High
	Mach-Zehnder interferometer [23]	3.7 MHz/°C	300 kHz	1800 s	-	3.6 km	0.08 °C	50 °C	Low
	Vernier effect [18]	210.25 kHz/°C	10 kHz	7200 s	-	231 m	0.048 °C	80 °C	High
	MRR [24]	10.02 GHz/°C	1.5 MHz	360 s	-	3 m	1.73 × 10^−4^ °C	1.2 °C	Medium
FBG	PVA-FBG-FP Filter [25]	1.8118 GHz/°C	180 kHz	600 s	-	-	0.2 °C	1 °C	Medium
	Cascaded TCFBG-FP/SMFBG-FP [26]	1.14 GHz/°C	200 kHz	1200 s	-	-	1.75 × 10^−4^ °C	1 °C	High
	Coarse sampling and Vernier [19]	222.84 kHz/°C	66.9 kHz	900 s	-	-	0.3 °C	70 °C	High
SBS	Single-passband MPF [17]	1.00745 MHz/°C	500 kHz	3000 s	>18 dBm	2.5 km	0.5 °C	340 °C	High
	Two fiber spools [27]	1.364 MHz/°C	400 kHz	500 s	12 dBm	20.5 km	0.3 °C	19.8 °C	High
	**FRM (Our Solution)**	**1.0609 MHz/°C**	**4.4 kHz**	**840 s**	**13.6 dBm**	5 km	**0.004** °C	**67** °C	**Low**
DFB-LD	Optical injection [28]	1.02 GHz/°C	80 MHz	600 s	-	1 km	0.08 °C	8 °C	High

## Data Availability

Data available upon reasonable request from the corresponding author.
